# Ursodeoxycholate Restores Biliary Excretion of Methotrexate in Rats with Ethinyl Estradiol Induced-Cholestasis by Restoring Canalicular Mrp2 Expression

**DOI:** 10.3390/ijms19041120

**Published:** 2018-04-09

**Authors:** Min Ju Kim, Yun Ju Kang, Mihwa Kwon, Young A. Choi, Min-Koo Choi, Hye-Young Chi, Hye Hyun Yoo, Chang-Koo Shim, Im-Sook Song

**Affiliations:** 1 Life Science Institute, Daewoong Pharmaceutical, Yongin, Gyeonggi-do 17028, Korea; yunijjan83@gmail.com (M.J.K.); hychi138@daewoong.co.kr (H.Y.C.); shimck@daewoong.co.kr (C.K.S.); 2 College of Pharmacy, Hanyang University, An-san, Gyeonggi-do 15588, Korea; yoohh@hanyang.ac.kr; 3 College of Pharmacy and Research Institute of Pharmaceutical Sciences, Kyungpook National University, Daegu 41566, Korea; yun-ju6895@nate.com (Y.J.K.); mihwa_k@naver.com (M.K); 4 College of Pharmacy, Dankook University, Cheon-an 31116, Korea; ayha06@gmail.com (Y.A.C.); minkoochoi@dankook.ac.kr (M.K.C.)

**Keywords:** ursodeoxycholate (UDCA), intrahepatic cholestasis, multidrug resistance-associated protein (Mrp) 2, methotrexate (MTX), biliary excretion clearance

## Abstract

The in vivo relevance of ursodeoxycholate (UDCA) treatment (100 mg/kg/day, per oral tid for 5 days before cholestasis induction followed by the same dosing for 5 days) on hepatic function was investigated in rats with 17α-ethinylestradiol (EE, 10 mg/kg, subcutaneous for 5 days)-induced experimental cholestasis. The bile flow rate and the expression level of hepatic multidrug resistance-associated protein 2 (Mrp 2) that were decreased in cholestasis were restored after UDCA treatment. Consistent with this, the biliary excretion clearance (CL_exc,bile_) of a representative Mrp2 substrate—methotrexate (MTX)—was decreased in cholestatic rats but was restored after UDCA treatment. Consequently, the plasma concentrations of MTX, which were increased by cholestasis, were decreased to control levels by UDCA treatment. Thus, the restoration of CL_exc,bile_ appears to be associated with the increase in Mrp2 expression on the canalicular membrane by UDCA treatment followed by Mrp2-mediated biliary excretion of MTX. On the other hand, the hepatic uptake clearance (CL_up,liver_) of MTX was unchanged by cholestasis or UDCA treatment, suggestive of the absence of any association between the uptake process and the overall biliary excretion of MTX. Since UDCA has been known to induce the expression of canalicular MRP2 in humans, UDCA treatment might be effective in humans to maintain or accelerate the hepatobiliary elimination of xenobiotics or metabolic conjugates that are MRP2 substrates.

## 1. Introduction

Ursodeoxycholate (UDCA), an endogenous hydrophilic bile salt, is predominant in bear bile and has been used as a traditional medicine for the treatment of jaundice. In 1989, the therapeutic efficacy of UDCA was re-demonstrated in the first clinical trials on patients with primary biliary cholangitis. UDCA has been marketed as a therapeutic for cholestasis and preventive drug for liver diseases [1,2]. Ursa^®^ (Daewoong Pharmaceutical Co. Ltd., Seoul, Korea), a single tablet with 100–300 mg of UDCA, has been marketed in Korea since 1961 to cure liver diseases, including cholestasis. Presently, UDCA is the most widely prescribed drug for the treatment of cholestasis and the only medicine approved by the US Food and Drug Administration to treat primary biliary cirrhosis [2].

The application of UDCA extends to the treatment of non-cholestatic liver diseases, owing to its multiple modes of action. This includes a reduction in the serum levels of toxic hydrophobic bile salts [3], the stimulation of the hepatobiliary excretion of xenobiotics via phase II and III detoxification processes [2,4], antioxidant activity against oxidative stress [2,5,6] and antiapoptotic activity through signaling pathways such as those involving the protein kinase C activator and mitogen-activated protein kinases (MAPKs) [2,7,8,9]. 

Studies have shown that UDCA pretreatment also prevents lipid peroxidation, increased cellular concentrations of reduced glutathione (GSH) [10] and superoxide dismutase and activation of nuclear factor-E2-related factor-2 (Nrf2) in rats [5,11,12]. GSH facilitates the hepatic elimination of xenobiotics by increasing GSH conjugation (phase II metabolism) and, consequently, enhancing the biliary excretion (phase III metabolism or detoxification) of GSH conjugates via canalicular membrane transporters, such as multidrug resistance-associated protein 2 (Mrp2) [13,14]. The greater affinity of GSH conjugates for Mrp2, as well as increased GSH conjugation [13,14,15,16,17], was proposed as mechanisms underlying the UDCA-mediated increase in the biliary excretion of certain xenobiotics. Mrp2 is involved in the biliary excretion of numerous anionic endobiotics and xenobiotics, as well as their glucuronide, sulfate [15,18] and GSH conjugates [13,14]. In combination with GSH, Mrp2 mediates the transport of amphipathic, unchanged drugs, such as cisplatin, vinblastine and sulfinpyrazone [19,20]. Therefore, Mrp2 is one of the most important canalicular transporters involved in the hepatic excretion of various xenobiotics and their metabolites into the bile. 

UDCA also increases the expression of Mrp2, Mrp3 and Mrp4 in rodents overexpressing Nrf2 [5] and bile salt export pump (Bsep) in rats with 17α-ethinylestradiol (EE)-induced cholestasis [21]. Therefore, UDCA treatment, which increases GSH conjugation and canalicular expression of efflux pump, may accelerate the hepatic elimination of endo- and xenobiotic Mrp2/Bsep substrates in a coordinated manner. 

In this study, we investigated the effects of UDCA treatment, in combination with canalicular transporter expression, hepatic and renal functions and protein binding of probe drug, on biliary probe drug excretion in rats with cholestasis. Methotrexate (MTX) was selected as the probe drug for Mrp2, because it is mainly excreted through the bile and urine in its parent form with limited metabolism, mainly by Mrp2 [22]. Cholestasis was experimentally induced through a subcutaneous (sc) injection of EE to male rats [23,24]. EE-induced cholestasis alters the expression and functions of drug transporters [25]. For instance, the mRNA expression of Na^+^-taurocholate co-transporting polypeptide (Ntcp), organic anion-transporting polypeptide (Oatp) 1a2 was downregulated in rats with EE-induced cholestasis [26]. Cholestasis reduced the canalicular expression of Bsep and Mrp2 [21,27,28]. Additionally, the internalization of canalicular Bsep and Mrp2 into the vesicular compartment of hepatocytes, followed by the reduction in the canalicular excretion of their substrates has been demonstrated in experimental cholestatic models induced by intravenous injections of estradiol-glucuronide and taurolithocholic acid [29,30]. Therefore, the effect of UDCA treatment on the expressional and functional changes of these transporters in cholestasis is worth investigating with respect to the biliary excretion (or detoxification) of their probe substrates.

## 2. Results

### 2.1. Effects of UDCA Treatment on the Hepatic Function of Rats with EE-Induced Cholestasis

**Figure 1:**
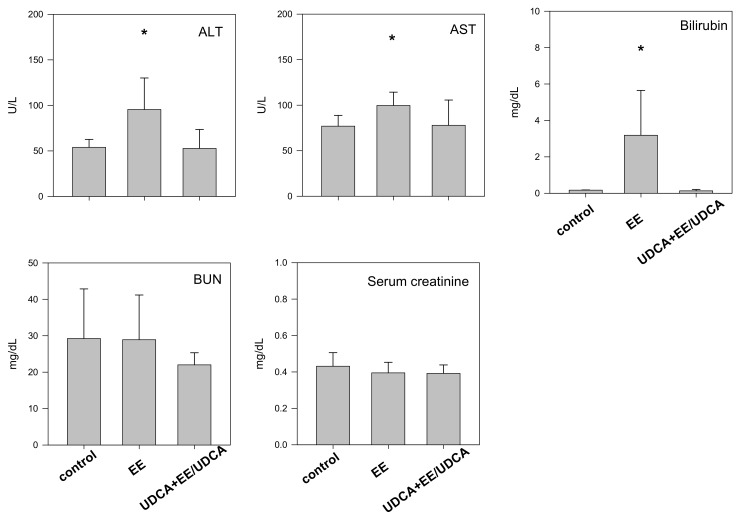
Biochemical parameters such as alanine aminotransferase (ALT), aspartate aminotransferase (AST), bilirubin, blood urea nitrogen (BUN) and serum creatinine level in control, EE and UDCA + EE/UDCA group. Each bar represents the mean ± SD from three different rats per group. * *p* < 0.05, significant compared with control group by student’s *t*-test.

Consecutive injections of EE resulted in about 12.8–15.2% decrease in the body weight and 65% decrease in the bile flow in rats from the EE group, suggestive of the induction of intrahepatic cholestasis mediated by EE injection (EE group in Table 1). UDCA treatment (UDCA + EE/UDCA group) resulted in a significant recovery of bile flow but not body weight (Table 1). The levels of alanine aminotransferase (ALT), aspartate aminotransferase (AST) and bilirubin were significantly elevated in the EE group, consistent with the induction of cholestasis and UDCA treatment restored the levels of these markers (Figure 1). This observation is consistent with the hypothesis that UDCA attenuates EE-evoked cholestasis. No change was observed in the level of blood urea nitrogen (BUN) and serum creatinine, indicative of the negligible impact of EE on kidney function.

### 2.2. Bile Salt Concentrations

As treatment with EE or UDCA may affect bile salt homeostasis, we evaluated the effect of EE and UDCA treatment on the concentration of bile salts in the plasma and bile at 12 h after the last injection of EE or UDCA + EE/UDCA. The concentration of total bile salt increased in the plasma samples from the EE group (Table 2); this increase may be associated with the decrease in the biliary excretion of salts. The concentration of total bile further increased in the UDCA + EE/UDCA-treated group (Table 2), owing to oral administration of UDCA. UDCA, glycine ursodeoxycholate (GUDCA) and taurousodeoxycholate (TUDCA)—conjugated metabolites of UDCA—were undetected in the plasma samples from the control and EE groups, while their levels increased to 29.8 ± 1.7, 2.6 ± 0.0 and 1.5 ± 1.3 μM, respectively, in the UDCA + EE/UDCA group (Table 2). 

### 2.3. Analysis of the mRNA Expression of Transporters in the Liver and the Kidney

**Figure 2:**
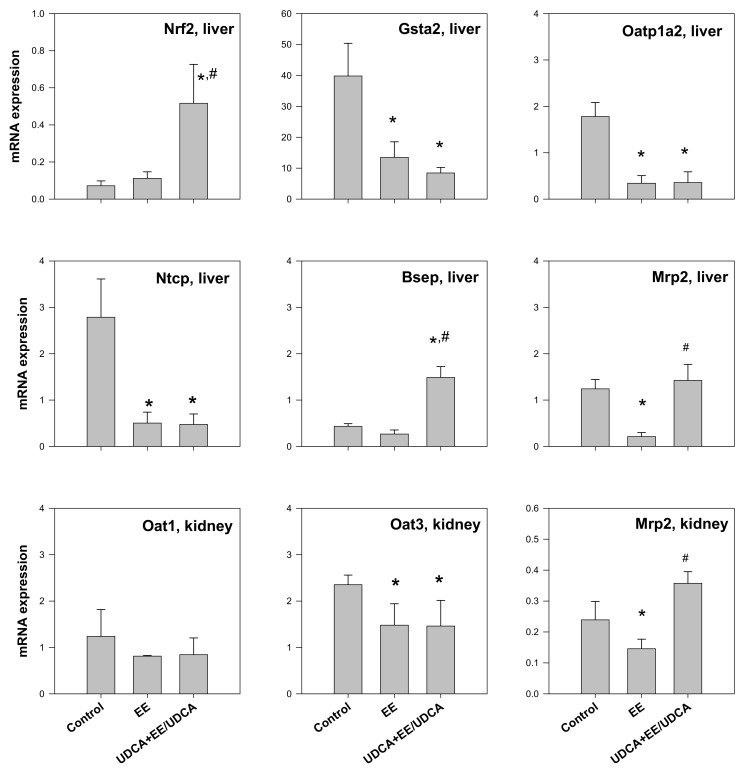
The mRNA expression level of Nrf2, Gsta2, Oatp1a2, Ntcp, Bsep and Mrp2 in the liver and the mRNA expression level of Oat1, Oat3 and Mrp2 in the kidney from control, EE and UDCA + EE/UDCA group. Each bar represents the mean ± SD from three different rats per group. * *p* < 0.05, significant compared with control group by student’s *t*-test. ^#^ *p* < 0.05, significant compared with EE group by student’s *t*-test.

As UDCA is known to regulate Nrf2-mediated redox regulation pathway [5], we measured the mRNA levels of Nrf2 and glutathione *S*-transferase A2 (Gsta2) in the liver of rats from each group. In addition, we evaluated the expression of transporter genes related to bile transport (Ntcp and Bsep). The level of Nrf2 slightly increased in the EE group and significantly increased in the UDCA + EE/UDCA group (Figure 2), consistent with the previous reports demonstrating the increase in Nrf2 pathway mediated by UDCA treatment [5,11]. Protein expression of Nrf2 was also consistent with mRNA levels of Nrf2 (Figure S1). On the other hand, the level of Gsta2 decreased in both the EE and UDCA + EE/UDCA groups, suggestive of the decreased role of the enzyme in both groups. The mRNA levels of Oatp1a2, Ntcp, and Mrp2 decreased in the EE group, as previously reported [26]. UDCA treatment failed to recover the levels of uptake transporters (Ntcp and Oatp1a2). However, Mrp2 level was restored to the control level following UDCA treatment. The level of Bsep was unchanged following EE treatment but increased to a level higher than that of the control group after UDCA treatment (Figure 2). Similar to mRNA expression of Ntcp, the protein levels of Ntcp significantly decreased in the EE and UDCA + EE/UDCA group. However, the protein levels of Bsep significantly reduced in the EE group but restored by the UDCA treatment (Figure S1). The reduced expression of Ntcp and Bsep in EE-induced cholestasis and restoration of Bsep by the UDCA treatment, consistent with the previous reports [21,26,27,28], could be underlying mechanisms for recovery of bile flow and total bile salts in the plasma and bile after the UDCA treatment (Table 1 and Table 2). 

The mRNA level of Oat1, Oat3 and Mrp2 in the kidney was evaluated. No change was observed in the level of Oat1 but a significant decrease in the level of Oat3 was reported in the EE and UDCA + EE/UDCA groups. The mRNA level of Mrp2 significantly decreased in the EE group but UDCA treatment significantly recovered Mrp2 level.

### 2.4. Pharmacokinetics of MTX

**Figure 3:**
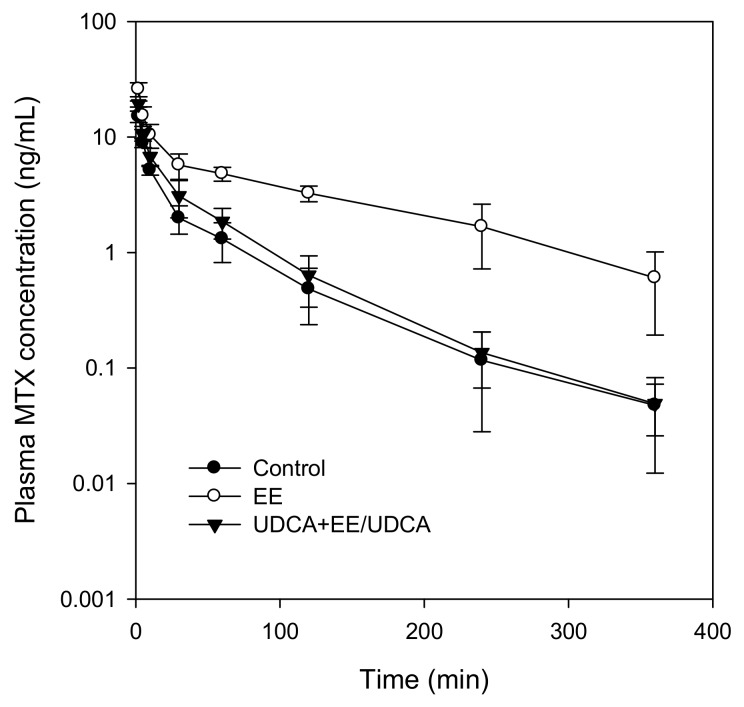
Plasma concentration-time profile of methotrexate (MTX) in control, EE and UDCA + EE/UDCA groups following intravenous injection of MTX at a dose of 3 mg/kg. Data point represents the mean ± SD from three or four different rats per group.

The compound MTX was intravenously administered at a single dose of 3 mg/kg and pharmacokinetic parameters related to the excretion of MTX were calculated (Table 3) from the plasma MTX concentration–time profiles (Figure 3). We observed a distinct increase in the plasma concentration of MTX, including the initial plasma concentration (C_0_), in EE-treated group (Figure 3) leading to a significant increase in the plasma AUC_6h_ and AUC_∞_ of MTX (Table 4). The biliary (CL_bile_) and urinary (CL_urine_) clearance, and consequently the CL_total_ of MTX, decreased in the EE-treated group (Table 3). UDCA treatment resulted in a significant recovery in the plasma concentration parameters such as C_0_, AUC_6h_, AUC_∞_, CL_total_, CL_bile_ and CL_urine_ (Table 3), demonstrating the restored biliary and urinary elimination of MTX.

### 2.5. Hepatic and Kidney Uptake of MTX

To elucidate the unit process responsible for the restored elimination of MTX by UDCA treatment, the uptake clearances from the plasma to the liver and kidney as well as the excretion clearances to the bile and urine were investigated. As unbound drugs could pass through the hepatobiliary and urinary excretion process, the plasma protein binding of MTX in each group was measured and found to be 68.05 ± 4.73%, 72.96 ± 3.10% and 72.45 ± 1.56% (mean ± SD, three rats per group) for the control, EE and UDCA + EE/UDCA group, respectively. No significant differences were observed in the values of three groups (*p* > 0.05 by the one-way ANOVA test). 

Table 4 summarizes MTX concentrations in the liver and kidney at 5 min after the intravenous administration and relevant plasma AUC_5min_ values of MTX. The uptake clearances of MTX into the liver (CL_up,liver_) and kidney (CL_up,kidney_) were calculated using the data presented in Table 5. No differences were observed in the plasma concentration and AUC_5min_ values in the liver among the experimental groups; hence, no significant differences were observed in CL_up,liver_ values of the groups (Table 4). Thus, cholestasis or UDCA treatment failed to influence MTX uptake into hepatocytes.

The concentration of MTX in the kidney was decreased either by cholestasis or UDCA treatment, leading to a significant decrease in CL_up,kidney_ for both experimental groups as compared with the control (Table 4). MTX acts as a substrate for both Oat1 and Oat3 in the kidney [31]. However, only the expression of Oat3 mRNA decreased in the EE and UDCA groups (Figure 2). Therefore, the significant decrease in CL_up,kidney_ of MTX (Table 4) appears to be attributable to the decrease in the expression of Oat3. 

**Figure 4:**
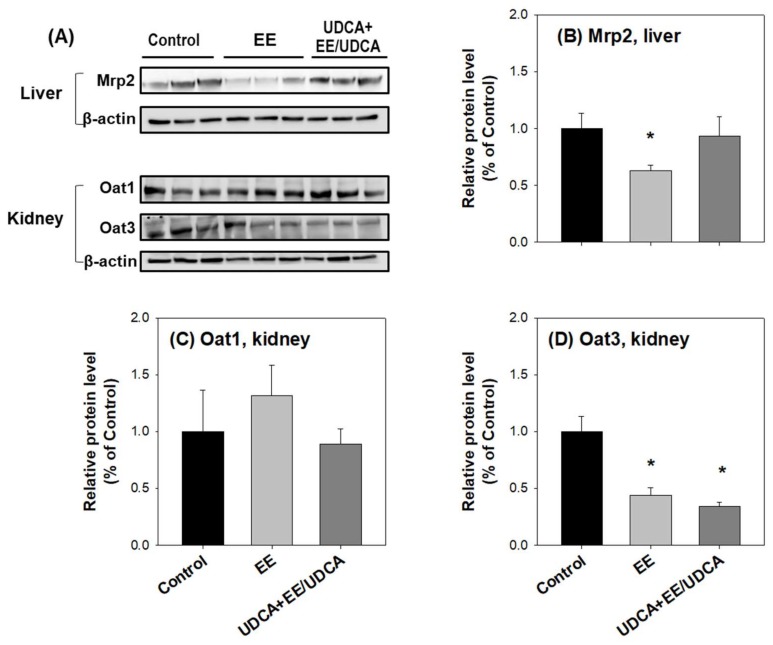
(**A**) Protein expression level of Mrp2 in the liver and levels of Oat1 and Oat3 in the kidney from control, EE and UDCA + EE/UDCA groups. (**A**) Lanes were loaded tissue lysates, which was prepared from three different rats per group. β-actin served as a loading control. Quantitative analyses of the western blot results are shown in (**B**–**D**). Each bar represents the mean ± SD. * *p* < 0.05, significant compared with control group by student’s *t*-test.

Consistent with the mRNA levels, no significant difference was observed in the protein level of Oat1 in the kidney among the experimental groups (Figure 4A,C); however, the protein level of Oat3 reduced in the kidney from the EE and UDCA groups as compared with that in the control group (Figure 4A,D). Thus, the decrease in the expression of Oat3 in the kidney accompanied with a decreased uptake may be responsible for the decreased MTX concentration in the kidney (Table 4). 

### 2.6. Biliary and Renal Excretion of MTX

The hepatobiliary excretion clearance (CL_exc,bile_) and urinary excretion clearance (CL_exc,urine_) of MTX were investigated in each experimental group to elucidate the contribution of two routes (i.e., hepatobiliary and urinary routes) to the overall elimination of MTX. MTX was administered via an intravenous bolus injection (1 mg/kg), followed by an infusion of MTX (0.6 mg/kg/h) for 6 h. 

The value of CL_exc,bile_ dramatically decreased in EE-induced cholestasis; however, UDCA treatment significantly recovered CL_exc,bile_ to the control level (Table 5). These results are consistent with the changes in the mRNA (Figure 2) and protein (Figure 4A,B) expression level of Mrp2 in the liver. 

No significant difference was observed in the values of CL_GF_, calculated from the renal clearance of creatinine (CL_cr_) and unbound fraction of MTX, between the three experimental groups (Table 5). The urinary excretion clearance of MTX (CL_exc,urine_), calculated by CL_urine_ − CL_GF_, decreased in the EE group but partially recovered in the UDCA group (Table 5). The phenotypic index of urinary excretion process, CL_exc,urine_, was consistent with the change in the expression level of Mrp2 in the kidney (Figure 2). Western blot analysis was performed for Mrp2 in the kidney; however, no signal was obtained, owing to the lower expression of Mrp2 in the kidney as compared with the liver. 

### 2.7. Correlation between the Expression Level of Transporters and In Vivo Kinetics of MTX

**Figure 5:**
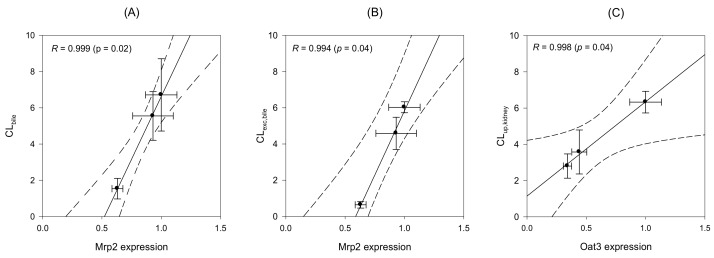
Correlation between Mrp2 protein expression and in vivo CL_bile_ (**A**) and CL_exc,bile_ (**B**) and between Oat3 protein expression and CL_up,kidney_ (**C**) of MTX. Mrp2 and Oat3 expression levels were recorded from the western blot analysis in Figure 4 and in vivo CL of MTX were selected from Table 3, Table 4 and Table 5. Lines were generated from the linear regression analysis and dotted lines represent 95% confidence interval from the geometric mean value.

Regression analysis was performed to elucidate the correlation between the changes in the expression of transporters (western blot results) and in vivo kinetics of MTX (in vivo clearance parameters). As shown in Figure 5A, Mrp2 protein expression in the liver was closely correlated with the in vivo biliary clearance (CL_bile_) (Figure 5A, *R* = 0.999, *p* = 0.02). Similar correlation was found for Mrp2 expression in the liver and biliary excretion clearance (CL_exc,bile_) of MTX (Figure 5B, *R* = 0.994, *p* = 0.04). These results strongly suggest the involvement of Mrp2 expression in the modulation of hepatobiliary excretion of MTX. However, no significant correlation was observed between Mrp2 expression and other parameters of in vivo MTX clearance (CL_up,liver_, CL_urine_ and CL_exc,urine_), suggestive of the absence of any association between Mrp2 and the process of hepatic uptake or urinary excretion of MTX. On the other hand, the renal expression of Oat3 was significantly correlated with CL_up,kidney_ of MTX (Figure 5C, *R* = 0.998, *p* = 0.04), indicative of the involvement of Oat3 expression in the modulation of renal uptake of MTX [31,32]. 

## 3. Discussion

The purpose of the present study is to elucidate the association of expressional changes of hepatic transporters by UDCA treatment with the change in the biliary excretion (or detoxification) of MTX in rats with cholestasis. UDCA was orally administered tid at a daily dose of 100 mg/kg for 10 days (UDCA alone for 5 days, followed by co-administration with EE for 5 days). The dose was selected based on the therapeutic dose of UDCA [33]. UDCA treatment for 10 days restored bile flow and biochemical abnormalities in the livers of rats with EE-induced cholestasis. During this treatment, the concentration of total bile salt in the plasma and bile was inversely changed by the treatment of EE and UDCA, demonstrating that decreased biliary secretion of bile salts is responsible for the increased plasma concentrations of bile salts in rats with cholestasis, consistent with a previous report [34]. When compared the steady-state plasma concentrations of UDCA and its metabolites TUDCA and GUDCA, UDCA was the main component among UDCA and the two conjugated metabolites. This suggested that UDCA itself contributed largely to the therapeutic effect of UDCA observed in this study. Additionally, the content of UDCA in the plasma increased dramatically from zero to 60% in this study. Altogether, the increased UDCA concentration and increased content of hydrophilic bile acid could play critical roles in facilitating bile flow and hepatic detoxification. Moreover, the steady-state concentration of UDCA observed in this study was comparable to that observed following therapeutic UDCA administration to humans (900 mg/day for 3 weeks) [35]. 

The expression of efflux transporters, such as Mrp2 and Bsep, was increased after UDCA treatment; however, the expression of uptake transporters, such as Ntcp, Oatp1a2, Oat1 and Oat3, was decreased by the treatment. Although the underlying mechanisms were not fully elucidated in the present study, transcriptional enhancement of Mrp2 and Bsep, via the enhanced cAMP-dependent protein kinase A (PKA) signaling and farnesoid X receptor (FXR) activation [36] or increased Nrf2 pathway signaling [5], might be responsible for the increased expression of the transporters. In addition, a good correlation was found between Mrp2 expression in the liver and the overall hepatobiliary excretion (CL_bile_) and canalicular excretion clearance (CL_exc,bile_) of MTX, suggesting that Mrp2-mediated canalicular excretion may regulate the elimination process and, thereby, normalize the pharmacokinetics of MTX, a Mrp2 substrate. 

In the clinical study, UDCA treatment (900 mg/day for 3 weeks) increases the expression of MRP2 on the bile canalicular membrane in the patients with early-stage primary biliary cholangitis and pregnancy-induced cholestasis [2,37]. Breast cancer resistant protein (BCRP) expression was also increased in patients with intrahepatic cholestasis of pregnancy treated with UDCA at a dose of 900 mg/day for 3 weeks [38]. Another UDCA administration (1 g/day for 3 weeks) to patients with gallstones stimulated the expression of MRP4, BSEP and MDR3 [39]. 

Taken together, UDCA treatment may offer benefits by facilitating the hepatobiliary elimination of various xenobiotics and their conjugated metabolites via increased expression of efflux transporters, including MRPs, BSEP, BCRP and MDR. Thus, UDCA could accelerate the phase III detoxification in the cholestasis in humans as well as in rats by controlling the expression and function of efflux transporters.

## 4. Materials and Methods

### 4.1. Materials

UDCA was obtained from Daewoong Pharmaceutical (Youngin, Korea), while EE, MTX and taurocholate were purchased from Sigma-Aldrich (St. Louis, MO, USA). GUDCA and TUDCA were supplied by Toronto Research Chemicals (Toronto, ON, Canada). 

### 4.2. Animals

Male rats (Sprague−Dawley, 220−250 g) were obtained from the Samtako Co. (Osan, Korea). All animal care and experimental procedures were approved by the Animal Care and Use Committee of Kyungpook National University (No. 2014-0131-1, 15 January 2015) and were carried out in accordance with the National Institutes of Health guidance for the care and the use of laboratory animals. Rats were housed for 1 week for acclimatization before

### 4.3. Treatment of Rats with EE and UDCA 

Based on the various preliminary experiments with different UDCA treatment regimens, rats were finally divided into three groups: control, EE (cholestasis) and UDCA + EE/UDCA group (Table 1). 

Rats from the control group were administered with a daily sc injection of propylene glycol (PG) at 10 a.m. for 5 consecutive days at a daily dose of 1 mL/kg, followed by an oral administration of 1% (*w*/*v*) carboxymethyl cellulose (CMC) suspension in tap water tid (9 a.m., 3 p.m. and 9 p.m.) at 2 mL/kg daily for subsequent 5 days.

Rats from EE group received a daily sc injection of PG solution of EE at 10 a.m. for 5 consecutive days at a dose of 10 mg/mL/kg [40]. CMC suspension (1%, *w*/*v*) without UDCA was then orally administered (2 mL/kg daily) tid (9 a.m., 3 p.m. and 9 p.m.) for subsequent 5 days.

Rats from UDCA + EE/UDCA group were orally administered with 1% (*w*/*v*) CMC suspension of UDCA tid (9 a.m., 3 p.m. and 9 p.m.) for 5 days at a daily UDCA dose of 100 mg/2 mL/kg, followed by a concomitant administration of EE and UDCA for another 5 days. EE was sc administered as a PG solution daily at 10 a.m. at a dose of 10 mg/mL/kg and UDCA was orally administered tid (9 a.m., 3 p.m. and 9 p.m.) as a 1% (*w*/*v*) CMC suspension at a daily dose of 100 mg/2 mL/kg.

The arterial plasma was collected from rats in each group at 12 h after the last treatment. The plasma sample was evaluated for biochemical parameters such as ALT, AST, bilirubin, bilirubin total, blood urea nitrogen (BUN) and serum creatinine level using assay kits from Young-Dong Diagnostics Co. (Seoul, Korea) in Seoul Clinical Laboratories (Yongin, Korea). 

The plasma protein binding of MTX was determined for each group using a rapid equilibrium dialysis kit (ThermoFisher Scientific Korea, Seoul, Korea) according to the manufacturer’s protocol. Briefly, 100 μL of plasma withdrawn from each group containing 1 μM MTX was added into the sample chamber of the semipermeable membrane (molecular weight cut-off 8000 Da) and 300 μL of phosphate buffered saline (PBS) was added to the outer buffer chamber. After 4 h incubation on a shaking incubator at 300 rpm at 37 °C, 50 μL aliquots from both sample and buffer chambers were taken and treated with equal volumes of fresh PBS and plasma, respectively, to match the sample matrices for liquid chromatography tandem-mass spectrometry (LC-MS/MS) analysis. Aliquots (100 μL) of matrix-matched samples were added to 500 μL of acetonitrile containing 2 ng/mL of propranolol (an internal standard). Samples were vortexed for 10 min and centrifuged at 13,200 rpm for 10 min. An aliquot (1 μL) of the supernatant was injected directly into LC-MS/MS system.

### 4.4. Isolation of Total RNAs and Real-Time Reverse-Transcription Polymerase Chain Reaction (RT-PCR) Analysis

Liver and kidney samples were collected from rats of each group at 12 h after the last treatment, were snap-frozen in liquid nitrogen and stored at −80 °C. Total RNA was extracted from 100 mg samples of the liver and kidney using RNAzol (Molecular Research Center Inc., Cincinnati, OH, USA). The purity of total RNA was confirmed by the absorption ratio between 260 nm and 280 nm and the concentration of total RNA was measured by UV spectrophotometry.

Real-time reverse transcription polymerase chain reaction was performed with the LightCycler 96 Real-Time PCR System (Roche, Madison, WI, USA). Each PCR program included a pre-incubation period at 95 °C for 10 min, followed by 45 cycles of denaturation at 95 °C for 10 s and annealing/extension at 60 °C for 10 s and 72 °C for 10 s. Probes used for RT-PCR are shown in Table 6. A relative quantitation of the mRNA level in the test tissue sample was made by measuring the threshold cycle (*C*_T_) values of target genes and hypoxanthine phosphoribosyltransferase 1 (*Hprt1*) house-keeping gene, which was used as an endogenous internal standard (IS) [41].

### 4.5. Western Blot Analysis

Total protein samples were prepared by homogenizing 100 mg samples of liver and kidney from each group with one volume of lysis buffer (150 mM NaCl, 1% NP-40, 0.5% sodium deoxycholic acid, 0.1% sodium dodecyl sulfate (SDS), 50 mM Tris, pH 7.5) for 10 min, followed by centrifugation at 13,200 rpm for 10 min. Protein samples (30–50 μg) were separated by SDS polyacrylamide gel electrophoresis (Bio-Rad, Hercules, CA, USA; 4–12% gradient gel) and transferred onto a nitrocellulose membrane (Bio-Rad). The membrane was blocked with 5% bovine serum albumin in Tris-buffered saline with Tween 20 (200 mM Tris, 1.37 M NaCl, 0.1% Tween 20, pH 7.6) for 1 h, followed by its overnight incubation at 4 °C with primary antibodies against Oat1 (1:200, Santa Cruz Biotechnology, Dallas, TX, USA), Oat3 (1:100, Santa Cruz Biotechnology), Mrp2 (1:1000, Abcam, San Francisco, CA, USA), Nrf2 (1:1000, Abcam), Ntcp (1:1000, Abcam), Bsep (1:1000, Abcam), and β-actin (1:1000, Santa Cruz Biotechnology). Following incubation, the membrane was rinsed thrice with TBST at 25 °C and treated with horseradish peroxidase-labeled anti-goat or anti-rabbit IgG antibody (Santa Cruz Biotechnology). Protein bands were visualized with an enhanced chemiluminescence system (Santa Cruz Biotechnology). The images were analyzed using an ImageQuant LAS 4000 Mini (GE healthcare Korea, Seoul, Korea).

### 4.6. Determination of Bile Salts in the Plasma and Bile

Total bile salt concentrations in plasma and bile samples were determined using an enzymatic-fluorometric assay with a slight modification to the method described by Choi et al. [42]. In brief, 100 μL of bile samples was collected 12 h after the last treatment of rats through the bile duct cannula, followed by the collection of 250 μL of blood samples from the abdominal artery. Aliquots (50 μL) of standard taurocholate solutions (5, 10, 25, 50, 100 and 200 μM), plasma samples and bile samples were added to 950 μL of the reaction buffer containing 1 mM β-nicotinamide adenine dinucleotide (β-NAD), 50 μU 3α-hydroxysteroid dehydrogenase (3α-HSD), 0.385 mM ethylenediaminetetraacetic acid (EDTA) and 760 mM Tris (pH 9.5) and incubated at 37 °C for 30 min. The reaction was stopped by adding 3 mL of ice cold water and the fluorescence of the samples was measured at 340 nm (excitation) and 465 nm (emission).

Concentrations of UDCA and its metabolites, GUDCA and TUDCA, in the plasma samples from the three groups were also measured according to the previously reported method [43]. Aliquots (50 μL) of the plasma sample were added to 250 μL of acetonitrile containing 0.2 ng/mL of UDCA-d5 (IS). Samples were vortexed for 10 min and centrifuged at 13,200 rpm for 10 min. An aliquot (1 μL) of the supernatant was injected directly into Agilent 6430 Triple Quad LC-MS/MS system (Agilent, Wilmington, DE, USA). 

UDCA, GUDCA and TUDCA were separated on a Synergi polar RP column (2.0 mm internal diameter × 150 mm length, 4 μm particle size) (Phenomenex, Torrance, CA, USA) with a mobile phase consisting methanol and water (65:35, *v*/*v*) containing 0.1% formic acid at a flow rate of 0.2 mL/min. 

Retention time was 6.3 min for TUDCA, 7.1 min for GUDCA, 13.0 min for UDCA and 13.0 min for UDCA-d5 (IS). Mass peak was monitored using selected reaction monitoring (SRM) at *m*/*z* 498.2 → 80.2 for TUDCA, *m*/*z* 448.1 → 74 for GUDCA, *m*/*z* 391.3 → 391.3 for UDCA and *m*/*z* 396.3 → 396.3 for UDCA-d5 in a positive ion mode. Calibration was applied on a standard curve in the range of 0.2–40 μM for TUDCA, GUDCA and UDCA. Linearity, accuracy, intraday precision and interday precision were found to be within the acceptance criteria.

### 4.7. Pharmacokinetics of MTX

The femoral arteries, femoral veins and bile duct of rats were cannulated with PE50 or PE10 polyethylene tubing (Jungdo, Seoul, Korea) under anesthesia with zoletil and lompun (50 and 5 mg/kg, respectively, intramuscular injection) and heparinized saline (10 U/mL) was used to prevent blood clotting. Pharmacokinetic studies were started at 12 h after the last treatment.

Methotrexate solution (3 mg/kg in PBS) was administered intravenously to rats. Blood samples were collected from the femoral artery at 0, 2, 5, 10, 15, 30, 60, 90, 120, 240 and 360 min after intravenous bolus injection and centrifuged at 13,200 rpm for 10 min. Bile samples were also collected every 60 min for up to 360 min through the bile cannula. Urine samples were collected for 6 h through urinary bladder. Aliquots of 50 μL of plasma, bile and urine samples were added to 250 μL of acetonitrile containing 2 ng/mL of propranolol (IS), followed by 10 min vortex-mixing and centrifugation at 13,200 rpm, an aliquot (1 μL) of the supernatant was injected directly into LC-MS/MS system.

The area under the plasma concentration–time curve from zero to infinity (AUC_∞_) was calculated by trapezoidal method and extrapolation method by dividing the terminal-phase rate constant with plasma concentration at the last time point [44]. Standard methods were used to calculate total clearance (CL_total_) and half-life by a non-compartmental analysis using WinNonlin (version 2.1, Pharsight, Mountain View, CA, USA). Urinary and biliary clearances of MTX (CL_urine_ and CL_bile_, respectively) were estimated by dividing the total amount of MTX excreted into urine and bile for 0–6 h with AUC_6h_. Glomerular filtration rate (GFR) was estimated from the creatinine clearance (CL_cr_), which was calculated by dividing the total creatinine amount in urine for 6 h by the mean plasma concentration of creatinine in each rat [45]. Creatinine concentration was assayed using kits from Young-Dong Diagnostics Co. (Seoul, Korea) from the service of Seoul Clinical Laboratories (Yongin, Korea).

### 4.8. Estimation of In Vivo Hepatic and Renal Uptake Clearances of MTX

To evaluate the in vivo hepatic (CL_up,liver_) and renal (CL_up,kidney_) uptake clearances of MTX, rats received a dose of 3 mg/kg of MTX via femoral vein catheter and blood samples (120 μL) were collected at 1, 2, 3, 4 and 5 min from the femoral artery. Animals were sacrificed at 5 min and the liver and kidney were dissected and weighed following an immediate and gentle wash with tissues soaked in ice-cold saline. The liver and kidney samples were homogenized with nine volumes of saline. The concentration of MTX in the plasma, liver and kidney was determined using LC-MS/MS system. Briefly, aliquots (50 μL) of plasma and 10% tissue homogenates were added to 250 μL of acetonitrile containing 2 ng/mL of propranolol (internal standard). The samples were vortexed for 10 min and centrifuged at 13,200 rpm for 10 min and an aliquot (1 μL) of the supernatant was injected directly into LC-MS/MS system. The values of CL_up,liver_ and CL_up,kidney_ were calculated by dividing the amount of MTX in the liver and kidney at 5 min with the plasma AUC from zero to 5 min. A period of 5 min was selected because the excretion of MTX from the liver and kidney was negligible for this period.

### 4.9. Estimation of In Vivo Biliary and Urinary Excretion Clearances of MTX

The femoral arteries, femoral veins and bile duct of rats were cannulated as described earlier. Rats received an intravenous bolus injection of MTX (1 mg/kg dissolved in 1 mL/kg PBS), followed by an intravenous infusion of MTX (0.6 mg/kg/h for 6 h, dissolved in 0.6 mg/0.5 mL PBS) to obtain steady-state concentrations of MTX in the liver. Bile was collected at an interval of 1 h for up to 6 h. Plasma and liver samples were immediately collected at 6 h. The concentrations of MTX in the plasma, liver, bile and urine samples were determined by LC-MS/MS method. In vivo biliary excretion clearance (CL_exc,bile_) was calculated by dividing the biliary excretion rate of MTX for 2–6 h (i.e., steady state of plasma MTX concentration) with the concentration of MTX in the liver at 6 h.

Urinary excretion clearance (CL_exc,urine_) of MTX was estimated using the following equation: CL_exc,urine_ = CL_urine_ − CL_GF_, where glomerular filtration CL of MTX (CL_GF_) was calculated by multiplying f_u_ (the unbound fraction of MTX in plasma) by GFR [46,47]. CL_urine_ was estimated by dividing the total amount of MTX excreted into urine for 0–6 h period with AUC_6h_.

### 4.10. LC-MS/MS Analysis of MTX

The concentration of MTX in each sample was analyzed using an Agilent 6430 Triple Quadrupole LC-MS/MS system equipped with an Agilent 1260 HPLC system (Agilent). The separation was carried out on a Synergi Polar RP column (2.0 mm internal diameter × 150 mm length, 4 μm particle size) (Phenomenex) using a mobile phase comprising water containing 0.1% (*v*/*v*) formic acid and acetonitrile containing 0.1% (*v*/*v*) formic acid at a ratio of 20:80. The flow rate was 0.25 mL/min. The operating parameters of the mass spectrometry were as follows: ion spray, 4000 V in negative mode; capillary temperature, 300 °C; vaporizer temperature, 300 °C; sheath gas pressure, 35 arbitrary units; auxiliary gas, 10 arbitrary units; and nitrogen gas flow rate, 10 L/min. The retention time was 1.8 min for MTX and 2.2 min for propranolol (IS). Quantitation was carried out by SRM at *m*/*z* 455.2 → 308.1 for MTX and *m*/*z* 260 → 116 for propranolol in the positive ionization mode and CE was 15 eV. Calibration was applied on a standard curve for MTX in the range of 0.02–50 μg/mL in plasma and kidney and liver homogenates, 0.5–1500 μg/mL in bile and 0.5–300 μg/mL in urine. Intra- and inter-day precision and accuracy had coefficients of variance of less than 15%.

## Figures and Tables

**Table 1:** Weight and bile flow change in rats following 17α-ethinylestradiol (EE) and ursodeoxycholate (UDCA) treatment.

Group	Treatment	Weight Change (%) ^(4)^	Bile Flow (mL/min) ^(4)^
Control	Vehicles only administration ^(1)^	0.48 ± 2.2	10.5 ± 2.3
EE (cholestasis)	sc injection of EE ^(2)^	−12.8 ± 2.4 *	3.7 ± 0.8 *
UDCA + EE/UDCA	Pretreatment with UDCA, followed by sc injection of EE and oral administration of UDCA ^(3)^	−15.2 ± 5.0 *	8.3 ± 1.7 ^#^

^(1^^)^ Rats were administered with a daily subcutaneous (sc) injection of propylene glycol at 10 a.m. for 5 consecutive days (1 mL/kg), followed by an oral administration of 1% (*w*/*v*) carboxymethyl cellulose (CMC) suspension in tap water tid (9 a.m., 3 p.m. and 9 p.m.) at 2 mL/kg daily for subsequent 5 days. ^(2^^)^ Rats were received a daily sc injection of propylene glycol solution of EE at 10 a.m. for 5 consecutive days at a dose of 10 mg/mL/kg. CMC suspension (1%, *w*/*v*) without UDCA was then oral administered (2 mL/kg daily) tid (9 a.m., 3 p.m. and 9 p.m.) for subsequent 5 days. ^(3^^)^ Rats were oral administered with 1% (*w*/*v*) CMC suspension of UDCA tid (9 a.m., 3 p.m. and 9 p.m.) for 5 days at a daily UDCA dose of 100 mg/2 mL/kg, followed by a concomitant administration of EE and UDCA for another 5 days according to the same methods in (2). This group was designated to represent the ‘UDCA-treated group.’ ^(4^^)^ Data were expressed as mean ± SD from three different rats per group. * *p* < 0.05, significant compared with control group by student’s *t*-test. ^#^ *p* < 0.05, significant compared with EE group by student’s *t*-test.

**Table 2:** Bile salt concentration in plasma and bile samples in control, EE and UDCA-treated groups.

Groups		Control (*n* = 3)	EE (*n* = 3)	UDCA + EE/UDCA (*n* = 3)
Total bile salt	Plasma (μM)	19.9 ± 1.7	32.5 ± 8.0 *	61.3 ± 27.6 *
	Bile (mM)	26.9 ± 6.2	16.7 ± 6.0 *	73.9^,^ ± 10.0 *
UDCA	Plasma (μM)	Not detected	Not detected	29.8 ± 1.7
GUDCA	Plasma (μM)	Not detected	Not detected	2.6 ± 0.0
TUDCA	Plasma (μM)	Not detected	Not detected	1.5 ± 1.3

Data were expressed as mean ± SD from three different rats per group. * *p* < 0.05, significant compared with control group by student’s *t*-test. ^#^ *p* < 0.05, significant compared with EE group by student’s *t*-test.

**Table 3:** Pharmacokinetic parameters of MTX following intravenous injection of MTX at a dose of 3 mg/kg.

Parameters/Groups		Control (*n* = 3)	EE (*n* = 4)	UDCA + EE/UDCA (*n* = 4)
C_0_	μg/mL	21.7 ± 5.0	36.9 ± 4.6 *	29.3 ± 2.1 *^,#^
AUC_6h_	μg·min/mL	320.5 ± 68.4	1167.8 ± 235.6 *	441.7 ± 104.8 ^#^
AUC_∞_	μg·min/mL	325.1 ± 71.9	1275.4 ± 341.9 *	445.9 ± 106.6 ^#^
t_1/2_	min	59.1 ± 14.9	105.1 ± 37.0	61.9 ± 8.6
CL_total_	mL/min/kg	9.56 ± 2.2	2.47 ± 0.6 *	7.02 ± 1.6 ^#^
CL_bile_	mL/min/kg	6.71 ± 2.0	1.54 ± 0.6 *	5.55 ± 1.3 ^#^
CL_urine_	mL/min/kg	3.69 ± 1.1	1.00 ± 0.2 *	2.02 ± 0.5 *^,#^

Data were expressed as mean ± SD from three or four different rats per group. * *p* < 0.05, significant compared with control group by student’s *t*-test. ^#^ *p* < 0.05, significant compared with EE group by student’s *t*-test. C_0_: initial plasma concentration. AUC_6h_ or AUC_∞_: Area under plasma concentration-time curve from zero to 6 h or infinity. t_1/2_: elimination half-life. CL_total_: total CL (Dose/plasma AUC). CL_bile_: biliary CL (Excreted amount in bile/plasma AUC). CL_urine_: urinary CL (Excreted amount in urine/plasma AUC).

**Table 4:** Hepatic and kidney uptake of MTX following intravenous injection of MTX at a dose of 3 mg/kg.

Parameters/Groups		Control (*n* = 3)	EE (*n* = 4)	UDCA + EE/UDCA (*n* = 4)
Plasma	AUC_5min_ (μg·min/mL)	45.5 ± 8.6	53.9 ± 8.4	50.4 ± 4.9
Liver	MTX concentration (μg/mL)	5.82 ± 1.3	5.06 ± 1.9	5.73 ± 1.6
	CL_up,liver_ (mL/min/kg)	4.69 ± 1.9	3.58 ± 1.4	3.84 ± 1.0
Kidney	MTX concentration (μg/mL)	38.0 ± 6.8	20.2 ± 4.0 *	16.8 ± 3.0 *
	CL_up,kidney_ (mL/min/kg)	6.33 ± 0.6	3.58 ± 1.2 *	2.80 ± 0.7 *

Data were expressed as mean ± SD from three or four different rats per group. * *p* < 0.05, significant compared with control group by student’s *t*-test. CL_up,liver_: liver uptake CL (MTX amount in the liver/plasma AUC). CL_up,kidney_: kidney uptake CL (MTX amount in the kidney/plasma AUC).

**Table 5:** Hepatobiliary and renal excretion of MTX following intravenous injection of 1 mg/kg and infusion of 0.6 mg/kg/h for 6 h of MTX.

Parameters/Groups	Control (*n* = 3)	EE (*n* = 4)	UDCA + EE/UDCA (*n* = 4)
CL_exc,bile_ (mL/min/kg)	5.63 ± 0.3	0.34 ± 0.2 *	4.09 ± 0.9 ^#^
CL_GF_ (mL/min/kg)	1.09 ± 0.02	0.81 ± 0.01	0.83 ± 0.01
CL_exc,urine_ (mL/min/kg)	2.60 ± 1.1	0.06 ± 0.2 *	1.20 ± 0.5 *^,#^

Data were expressed as mean ± SD from three or four different rats per group. * *p* < 0.05, significant compared with control group by student’s *t*-test. ^#^ *p* < 0.05, significant compared with EE group by student’s *t*-test. CL_exc,bile_: biliary excretion CL (MTX excretion rate into bile/MTX concentration in the liver at steady state. CL_GF_: glomerular filtration CL (creatinine CL (GFR) × unbound fraction of MTX). CL_exc,urine_ urinary excretion CL (CL_urine_ − CL_GF_).

**Table 6:** Primer sequences of target genes used for real-time PCR.

Gene	Reference	Primer	Sequence	Length	Product Size
*Nrf2*	NM_031789	Forward	5′-agcatgatggacttggaattg-3′	21 bp	78 bp
		Reverse	5′-cctccaaaggatgtcaatcaa-3′	21 bp	
*Gsta2*	NM_017013	Forward	5′-tgacctctttccctctgctg-3′	20 bp	78 bp
		Reverse	5′-caggctgcaggaacttcttc-3′	20 bp	
*Abcc2* (*Mrp2*)	NM_012833	Forward	5′-cggtgcactttaacatctgc-3′	20 bp	92 bp
		Reverse	5′-tctcctcgcgcttctgttac-3′	20 bp	
*Slco1a2* (*Oatp1a2*)	NM_131906	Forward	5′-caagaaagctgcacacttagca-3′	22 bp	60 bp
		Reverse	5′-aggaaagacagaaggtactcagaca-3′	25 bp	
*Slc10a1* (*Ntcp*)	NM_017047	Forward	5′-aagggggacatgaacctca-3′	19 bp	66 bp
		Reverse	5′-catcatgcccaaggcact-3′	18 bp	
*Abcb11* (*Bsep*)	NM_031760	Forward	5′-cggccatgactgatttaagc-3′	20 bp	60 bp
		Reverse	5′-atagctcctgccaaacttgc-3′	20 bp	
*Slc22a6* (*Oat1*)	NM_017224	Forward	5′-caagcctcagccatggag-3′	18 bp	91 bp
		Reverse	5′-aggcaaagctagtggcaaac-3′	20 bp	
*Slc22a8* (*Oat3*)	NM_031332	Forward	5′-ttggatggctggatctacaa-3′	20 bp	65 bp
		Reverse	5′-ctgcacaccaagtcccact-3′	19 bp	

## Supplementary Materials

Supplementary materials can be found at http://www.mdpi.com/1422-0067/19/4/1120/s1.

## Abbreviations

UDCA	ursodeoxycholate
GUDCA	glycine ursodeoxycholate
TUDCA	taurousodeoxycholate
EE	17α-ethinylestradiol
Mrp2/MRP2	multidrug resistance-associated protein 2
MTX	methotrexate
GSH	reduced gluthathione
Nrf2	nuclear factor-E2-released factor 2
Ntcp	Na^+^-taurocholate co-transporting polypeptide
Oat	organic anion transporter
Oatp	organic anion transporting polypeptide
Bsep	bile salt export pump
ALT	alanine aminotransferase
AST	aspartate aminotransferase
PG	propylene glycol
CMC	carboxymethyl cellulose
BUN	blood urea nitrogen
PBS	phosphate buffered saline
LC-MS/MS	liquid chromatography tandem-mass spectrometry
RT-PCR	real-time reverse-transcription polymerase chain reaction
Hprt	hypoxanthine phosphoribosyltransferase
IS	internal standard
SDS-PAGE	sodium dodecyl sulfate-polyacrylamide gel electrophoresis
β-NAD	β-nicotinamide adenine dinucleotide
3α-HSD	3α-hydroxysteroid dehydrogenase
EDTA	ethylenediaminetetraacetic acid
AUC	area under the plasma concentration–time curve
CL_total_	total clearance
CL_urine_	urinary clearance
CL_bile_	biliary clearance
GFR	glomerular filtration rate
CL_cr_	creatinine clearance
CL_GF_	glomerular filtration clearance
CL_up,liver_	liver uptake clearance
CL_up,kidney_	kidney uptake clearance
CL_exc,bile_	biliary excretion clearance
CL_exc,urine_	urinary excretion clearance
