# Supplementary material for: Ursodeoxycholate Restores Biliary Excretion of Methotrexate in Rats with Ethinyl Estradiol Induced-Cholestasis by Restoring Canalicular Mrp2 Expression
Source: Int J Mol Sci. 2018 Apr 9;19(4):1120. doi: 10.3390/ijms19041120 (PMC5979538; doi:10.3390/ijms19041120)
Supplement: Supplementary file 1 [file ijms-19-01120-s001.pdf]

International Journal of Molecular Sciences

MDPI

Supplementary Materials: Ursodeoxycholate Restores Biliary Excretion of Methotrexate in Rats with Ethinyl Estradiol Induced-Cholestasis by Restoring Canalicular Mrp2 Expression

Min Ju Kim [1,2], Yun Ju Kang [3], Mihwa Kwon [3], Young A. Choi [4], Min-Koo Choi [4], Hye-Young Chi [1], Hye Hyun Yoo [2], Chang-Koo Shim [1] and Im-Sook Song [3,*]

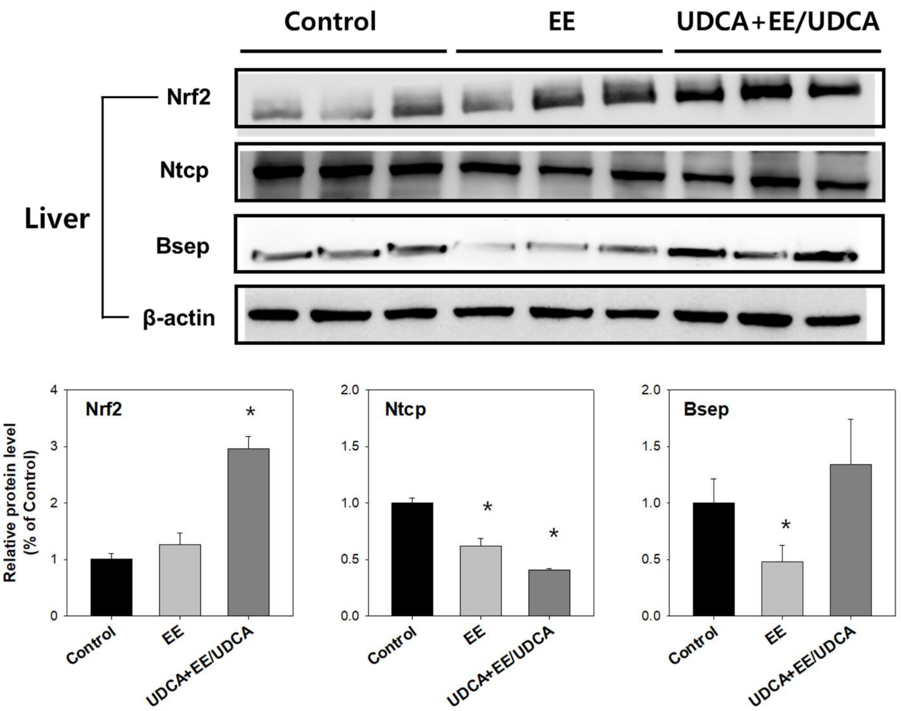

Figure S1. Protein expression level of Nrf2, Ntcp, and Bsep in the liver from control, EE and UDCA + EE/UDCA groups. Lanes were loaded liver lysates, which was prepared from three different rats per group. β-actin served as a loading control. Quantitative analyses of the western blot results are shown in the lower panel. Each bar represents the mean ± SD. * $p < 0.05$, significant compared with control group by Student's *t*-test.
